# Study on cerium-doped nano-TiO_2_ coatings for corrosion protection of 316 L stainless steel

**DOI:** 10.1186/1556-276X-7-227

**Published:** 2012-04-19

**Authors:** Suning Li, Qian Wang, Tao Chen, Zhihua Zhou, Ying Wang, Jiajun Fu

**Affiliations:** 1 School of Chemical Engineering, Nanjing University of Science and Technology, Nanjing, 210094, China; 2 Business School, China University of Political Science and Law, Beijing, 102249, China; 3 State Key Laboratory of Electronic Thin Film and Integrated Devices, School of Microelectronics and Solid-state Electronics, University of Electronic Science and Technology of China, Chengdu, 610054, China

**Keywords:** Nano-TiO_2_ coating, Cerium ion doping, Corrosion protection

## Abstract

Many methods have been reported on improving the photogenerated cathodic protection of nano-TiO_2_ coatings for metals. In this work, nano-TiO_2_ coatings doped with cerium nitrate have been developed by sol–gel method for corrosion protection of 316 L stainless steel. Surface morphology, structure, and properties of the prepared coatings were investigated by X-ray diffraction, X-ray photoelectron spectroscopy, scanning electron microscopy and energy dispersive X-ray spectroscopy. The corrosion protection performance of the prepared coatings was evaluated in 3 wt% NaCl solution by using electrochemical techniques in the presence and absence of simulated sunlight illumination. The results indicated that the 1.2% Ce-TiO_2_ coating with three layers exhibited an excellent photogenerated cathodic protection under illumination attributed to the higher separation efficiency of electron–hole pairs and higher photoelectric conversion efficiency. The results also showed that after doping with an appropriate concentration of cerium nitrate, the anti-corrosion performance of the TiO_2_ coating was improved even without irradiation due to the self-healing property of cerium ions.

## Background

The applications of TiO_2_ coatings for photocathodic protection of steels under ultraviolet (UV) illumination have attracted considerable interest because of their unique optical and electrical properties [1-7]. The principle of photocathodic protection lies in the fact that when a metal coated with a thin TiO_2_ coating is exposed to UV irradiation, electron–hole pairs are generated in the TiO_2_ coating [8]. The photogenerated electrons transfer to the metal substrate thereby making its electrode potential more negative than its corrosion potential. Especially, in this photoelectrochemical anticorrosion system, the TiO_2_ coating functions as a non-sacrificial anode when used for cathode protection of steel.

However, the practical application of this technology has been somewhat limited due to the high recombination of photogenerated e^−^-h^+^ pairs. Meantime, the pure TiO_2_ coating cannot provide photogenerated cathodic protection in dark condition. To solve the above-mentioned problems, there have been a number of studies related to doped TiO_2_ with materials such as WO_3_[9-12], SnO_2_[13-15], MoO_3_[16], phosphotungstic acid [17], and Ni(OH)_2_[18]. The semiconductor with a different energy level from TiO_2_ can store excess electrons during irradiation. Subsequently, the stored electrons can be released in the dark, thus maintaining a more negative potential and offering a cathodic protection. However, this protection system cannot work well during a long-time dark condition. Thus, the investigation of the TiO_2_ coating as protective materials for steels without the photo effect is of considerable practical interest. Up to now, there are few reports on the corrosion behavior of the modified nano-TiO_2_ coatings without photo effect. In 2007, Yun et al. have revealed that N-doped TiO_2_ coatings show a remarkable improvement in corrosion protection without the photo effect due to their compact structure and hydrophobic property [19].

In order to combine the photogenerated cathodic protection performance and the self-protection of TiO_2_ coating without the photo effect, it is highly required to find a new approach to improve the TiO_2_ coating as an excellent protective coating for metals. Herein a sol–gel method is used to modify nano-TiO_2_ coating with cerium ion by adding the concentrated solution of cerium nitrate. The cerium ion-doped TiO_2_ coating is taken into consideration for two reasons; on one hand, the Ce-doped TiO_2_ has widely been used as a photocatalyst to enhance the photocatalytic activity of TiO_2_ in the visible region [20-22] because it yields a large red shift compared to pure TiO_2_ and suppresses electron–hole recombination with electron trapping at Ce^4+^. We make use of this advantage in the field of photocatalysis to verify its improvement in the photogenerated cathodic protection for metals. On the other hand, cerium nitrate is known as a corrosion inhibitor in an aqueous aggressive medium. The corrosion inhibition properties of cerium nitrate have been widely discussed in literatures [23-25]. It is generally accepted that cerium ions lead to the precipitation of cerium oxides or hydroxides that suppress the corrosion reactions in the defects. Moreover, the incorporation of corrosion inhibitors into the sol–gel films can improve the protective ability of the coatings [26,27]. We expect that the cerium ions doped in the TiO_2_ coating can also enhance the self-protection property of the coating based on its self-healing effect without photo effect.

## Methods

### Preparation of Ce-TiO_2_ sol

All reagents were purchased from Chinese Chemical Reagent Company (Tianjin Xibeier International Co. Ltd., Tianjin, China). The cerium ion-doped TiO_2_ sol was prepared by a sol–gel process with the following procedure: tetra-*n*-butyl titanate (Ti(O-*n*-Bu)_4_) used as titania precursor was added into the mixture solution of ethanol (EtOH) and triethanolamine (TEA) at room temperature, which was denoted as solution A**.** The molar ratio of EtOH: TEA was 23:1. Ce(NO_3_)_3_·6H_2_O was dissolved in H_2_O and then mixed with EtOH, denoted as solution B. The molar ratio of Ti (O-*n*-Bu)_4_:H_2_O was 1:9, and H_2_O:EtOH was 1:2. Then solution B was slowly added into solution A under vigorous stirring. Later, acetylacetone was dropped into the mixed solution with further stirring for 3 h at room temperature. The obtained transparent sol was aged for 4 days before using. The sols with different cerium ion content were prepared according to the nominal atomic ratio of Ce/Ti, such as 0.2%, 1.2%, and 2.2%. Besides, the pure TiO_2_ sol was also prepared for a comparison.

### Preparation of Ce-TiO_2_ coatings

316 L stainless steel sheets were used as the metal substrate. Before coating, the surface of the metal was ground with grade 400–2000 emery papers gradually, then degreased in acetone, ethanol, and distilled water for 10 min, respectively. The coating was formed on the steel substrate by the dip-coating method. Firstly, the 316 L stainless steel sheet was immersed in the sol for 3 min and withdrawn at a speed of 12 mm/min. After natural drying in an air flow, the sample was dried in the oven at 100°C for 10 min. In order to obtain coatings with different thickness, the above-mentioned operation was repeated for several times. Then the sample was heat-treated in a muffle furnace at 250°C for 30 min, and at 500°C for 60 min. Finally, the coated sample was immersed in boiling water for 10 min, and thermally treated in the muffle oven again at 500°C for 10 min to eliminate the crack defects in the coating.

### Analysis of the prepared coatings

The crystal structure of the Ce-TiO_2_ coatings was characterized by X’ pert pro X-ray diffraction system (PANalytical BV, Almelo, The Netherlands) with Cu radiation (λ = 1.5406 Å) at a scanning rate of 1° min^−1^ for 2*θ* ranging from 10° to 80°.

The X-ray photoelectron spectra were obtained with a Multi Lab 2000VG spectrometer (VG Thermo Scientific Ltd., East Grinstead, UK) using 300 W Mg Kα radiation. The air pressure in the vacuum chamber was below 10^−8^ Pa. The binding energy was corrected by taking the C 1 s level as 284.8 eV. Quantitative analysis was carried out using the sensitivity factors supplied with the instrument.

The surface morphology of the sol–gel coatings was studied by scanning electron microscope (SEM) using LEO 1530 microscope (LEO Electron Microscopy Ltd., Cambridge, England) with 15 kV accelerating voltage of electron beam. Elemental chemical analysis of the coatings was performed by energy dispersive x-ray spectroscopy (EDX) connected to the SEM.

### Electrochemical tests

Each electrochemical experiment was performed at room temperature in 3 wt% NaCl solution in an electrochemical cell equipped with a quarts glass window by using PAR2273 electrochemical measurement system (EG & G Inc., Akron, OH, USA). The test cell included a saturated calomel electrode as the reference electrode, a platinum counter electrode, and the Ce-TiO_2_-coated 316 L stainless steel used as the working electrode. The working electrode was embedded in epoxy resin and only remained an exposed area (0.25 cm^2^) for testing. A 500 W Xe lamp was used as the light source to simulate the sunlight illumination. The variation of the open circuit potential (OCP) and the polarization curves were investigated in the presence and absence of light illumination. The Tafel curves were measured between ±250 mV at the open circuit potential at the rate of 1 mV/s and started after 30 min immersion of the samples in the NaCl solution. The electrochemical impedance spectrum (EIS) was measured with a frequency ranging from 0.1 up to 100 kHz, amplitude 10 mV at OCP. The impedance plots were fitted using Zsimpwin software (EChem Software Company, MI, USA) with compatible equivalent circuits which simulated the corrosion behavior of the coated metal. All the experiments were carried out in a Faraday cage to avoid external electromagnetic interference.

## Results and discussion

### Photo-electrochemical performance

**Figure 1:**
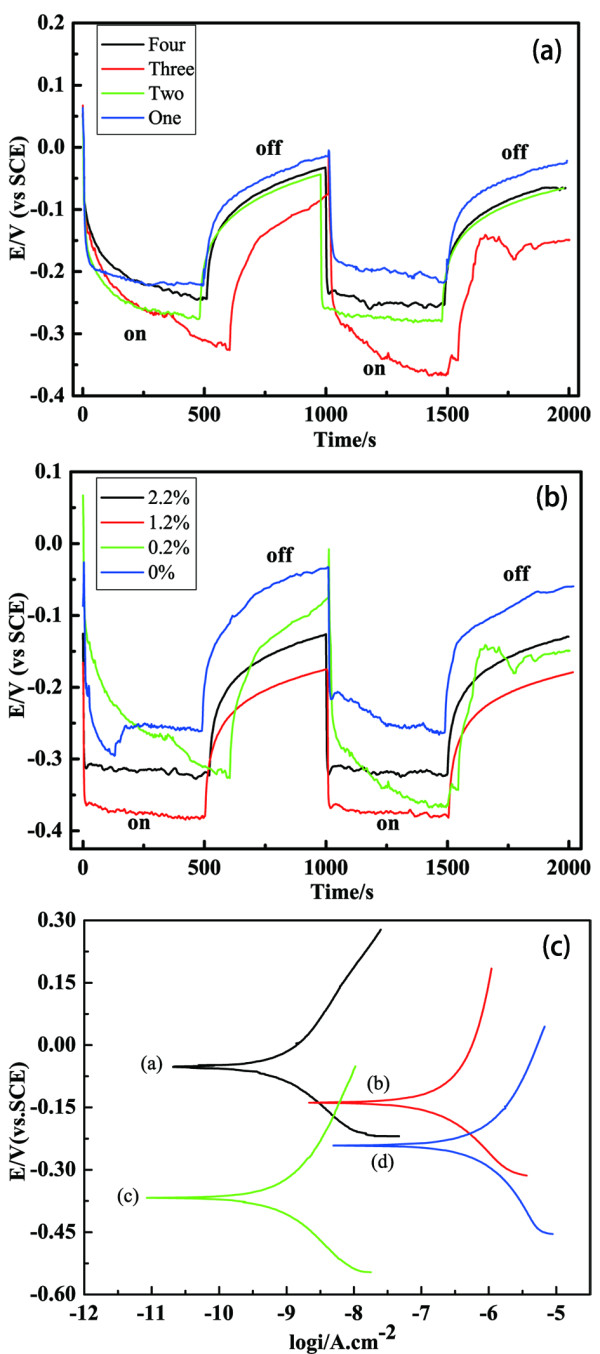
**Variation of the OCP.** At (**a**) for the 0.2% Ce-TiO_2_ coatings with different layers under illumination, (**b**) for the three-layer Ce-TiO_2_ coatings with different cerium ion contents under illumination, and (**c**) Tafel curves of pure TiO_2_ coating and 1.2% Ce-TiO_2_ coating under illumination and in the dark. *Line a*: 1.2% Ce-TiO_2_ in the dark, *line b*: pure TiO_2_ in the dark; *line c*: 1.2% Ce-TiO_2_ under illumination; *line d*: pure TiO_2_ under illumination.

Firstly, the influence of coating thickness on photogenerated cathodic protection for the metal substrate has been studied. Figure 1a describes the variation of the OCP for 0.2% Ce-TiO_2_ coating samples with different coating thickness. It is shown that under illumination, the OCP drops immediately to a more negative value than the corrosion potential of 316 L stainless steel (−180 mV). The metal substrate is maintained under a photogenerated cathode protection condition, which is caused by the sudden creation of photogenerated electron–hole pairs in the cerium ion-doped TiO_2_ coating. After a while, the OCP shifts slowly and tends to have a relatively steady value, owing to the balancing rate between creation and deletion of photogenerated electrons. As coating thickness increases, the stable potential negatively shifts. It is obvious that the OCP for the three-layer sample reaches the most negative value. Since the adsorption of photons by the Ce-TiO_2_ coating increases with its thickness, it is saturated when the coating is thicker than the light penetration depth. So the Ce-TiO_2_ coating with three layers exhibits the highest photoelectric conversion efficiency. After the illumination is stopped, the OCP of each sample rises quickly and coatings cannot provide a cathode protection for the metal substrate.

Secondly, the effect of cerium ion doping content has been investigated. Figure 1b shows the variation of the OCP for three-layer Ce-TiO_2_ coatings with different cerium ion contents. It is displayed that the OCP negatively shifts initially, and the metal substrate is maintained under a photogenerated cathode protection condition. When the light is turned off, the OCP rises quickly in a few minutes and reaches to a value more positive than the corrosion potential of 316 L stainless steel. It cannot retain the cathode protection in the dark. Compared to the undoped TiO_2_ coating sample, the doped samples reach a more negative photopotential under illumination. The results indicate that cerium ion doping can improve the photogenerated cathodic protection of nano-TiO_2_ coating. Moreover, it can be seen that an optimal cerium ion doping content exists, which is the 1.2% Ce-TiO_2_ coating sample. The effect of cerium ion on improving the photogenerated cathodic protection property of nano-TiO_2_ coating is discussed in the section of X-ray photoelectron spectroscopy (XPS) analyses for more details.

Figure 1c shows the polarization curves for pure TiO_2_ and 1.2% Ce-TiO_2_-coated 316 L stainless steel in the presence and absence of light illumination in 3 wt% NaCl solution. The polarization curves of cerium ion-doped TiO_2_ coating shifts to more negative values under illumination in comparison with that of pure TiO_2_ coating. This phenomenon conforms to the variation of OCP for both samples, which has been explained in the former discussion. In the presence of illumination, the anodic current for each sample becomes higher than in the dark, which is due to the photogenerated electrons excited from the coating to the metal substrate. Compared with the undoped coating, the Tafel curve for the doped coating exhibits a positive corrosion potential in the absence of illumination, which can be correlated with the higher corrosion resistance of the coating. Moreover, the 1.2% Ce-TiO_2_ coating displays a much lower anodic current as compared to the undoped coating in the dark condition. It is proved that cerium ion-doped TiO_2_ coating provides an enhanced corrosion protection either under illumination or even in the dark.

### XPS analyses

**Figure 2:**
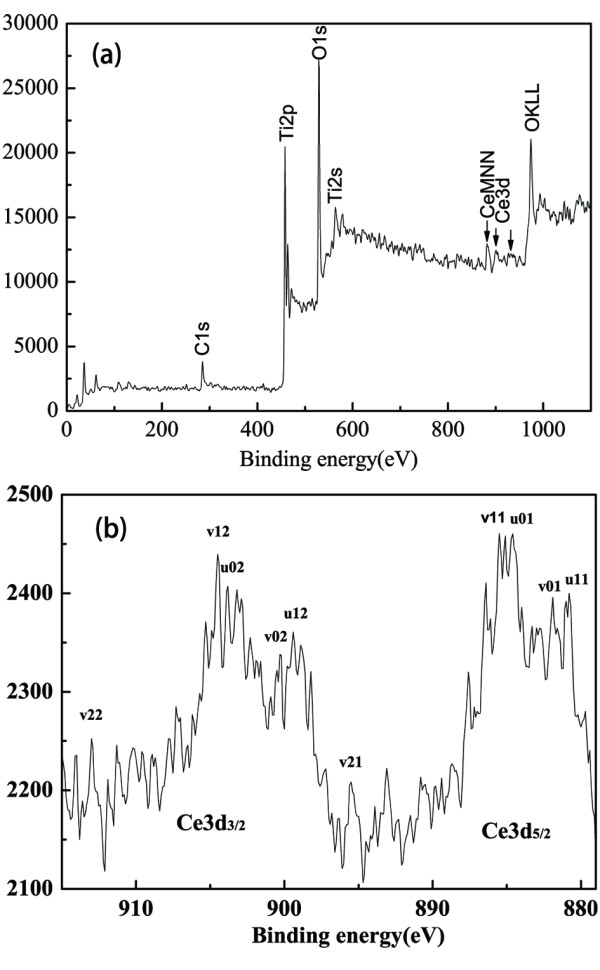
**XPS spectra of (a) 1.2% Ce-TiO**_**2**_**coating and (b) Ce 3d region for 1.2% Ce-TiO**_**2**_**coating.** KLL and MNN are the Auger group for O and Ce, respectively.

Survey spectrum for 1.2% Ce-TiO_2_ coating shows the presence of Ce, C, Ti, and O, which is depicted in Figure 2a. The Ce 3d XPS spectrum of 1.2% Ce-TiO_2_ coating sample is shown in Figure 2b. The spectrum basically denotes a mixture of Ce^3+/4+^ oxidation states giving rise to several peaks, indicating the coexistence of Ce^3+^ and Ce^4+^ in the coating.

Based on the spectral literature [28,29], the Ce 3d spectrum is rather complex and can be assigned 3d_3/2_ spin-orbit states and 3d_2/5_ states. The peaks labeled ‘*u*’ and ‘*v*’ corresponded to Ce^3+^ and Ce^4+^ in ceria, respectively, in which the v01, v11, and v21 at 881.9, 886.4, and 895.5 eV represented Ce^4+^, while the u01 and u11 at 885.5 and 880.8 eV represented Ce^3+^ in the Ce 3d_5/2_ spin-orbit split doublet. Correspondingly, the v02, v12, and v22 at 899.4, 904.5, and 913.0 eV represented Ce^4+^, while the u02 and u12 at 902.9 and 898.2 eV represented Ce^3+^ in the Ce 3d_3/2_ spin-orbit split doublet.

The Ce^4+^ ion is superior in the capability of trapping conduction band electrons under irradiation, and then forms its reduced state Ce^3+^. Moreover, the electrons trapped in the Ce^4+^/Ce^3+^ site are subsequently transferred to the metal substrate thereby making its electrode potential more negative than its corrosion potential. So the Ce^4+^ can act as the scavenger of electron and influence the photogenerated cathodic protection property by suppressing the recombination of the electron–hole pairs. However, it becomes the recombination center of electron–hole pairs when cerium ion-doped concentration increases further, because doping concentration also affects the thickness of the space charge layer (*W*). With an increased doping concentration, the space charge layer gets narrower. Therefore, the electron and the hole can be separated efficiently, and the lifetime of the photogenerated electron–hole pairs gets extended. When the cerium ion doping concentration is lower than the optimal concentration, the photogenerated electron–hole pairs cannot be separated most efficiently because there are not enough traps to capture the photo-induced electron. Only if the value of *W* approximates that of the penetration depth of the light into the solid, all the photons absorbed generate electron–hole pairs that are separated efficiently [30], and considerable electrons can finally be transferred to the metal substrate, making the photopotential reduced to the most negative value. Consequently, it is understandable that an optimal cerium ion doping content exists, which has also been shown in the section of photopotential variation with time.

### XRD measurements

**Figure 3:**
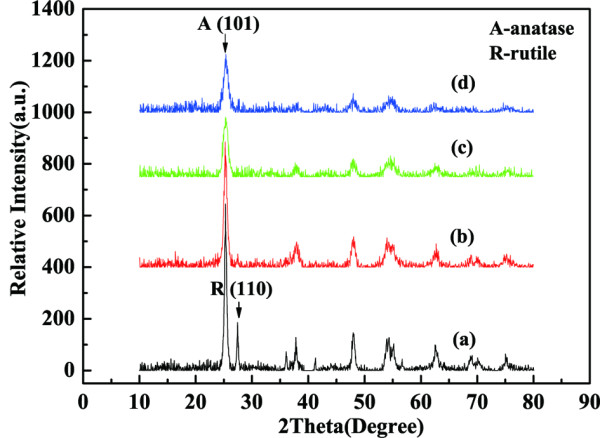
**XRD spectra of different Ce-TiO**_**2**_**coatings.** (**a**) pure TiO_2_, (**b**) 0.2% Ce-TiO_2_, (**c**) 1.2% Ce-TiO_2_, and (**d**) 2.2% Ce-TiO_2_.

Figure 3 shows the X-ray diffraction (XRD) spectra for Ce-TiO_2_ coatings with different cerium ion doping contents. All the coatings doped with cerium have a similar pattern dominated by anatase phase, while the pure TiO_2_ sample contains rutile phase besides anatase phase, which indicates that cerium ion doping hinders phase transformation from anatase to rutile. With the increase of cerium ion content, the main anatase peak (101) gets broader and the relative intensity is decreased, so the average crystallite sizes calculated using Scherer equation decrease as well. Moreover, the crystal lattice aberrance can be obtained using the following equation [31]:

(1) $$\varepsilon =\frac{\Delta d}{d}=\frac{\beta }{4tg\theta }$$

Where *ε* is the crystal lattice aberrance, *d* the space between crystal faces, *Δd* the error of the space between crystal faces, *β* the peak width at half height of diffraction peak, and *θ* is the half diffraction angle.

The results depicted in Table 1 revealed that due to doping with cerium ion, the crystal lattice expansion could suppress the growth of anatase microcrystal and thereby, the crystallite size was decreased. The degree of crystal lattice aberrance was also enhanced with the decreasing of the crystallite size, resulting in the creation of oxygen vacancies which could be act as the scavenger of holes. So doping with cerium ion could reduce the recombination of electron–hole pairs and improve the photogenerated cathodic protection property.

**Table 1:** **Crystal parameters of Ce-TiO**_**2**_**coatings**

**Samples**	**TiO**_**2**_	**0.2%Ce-TiO**_**2**_	**1.2%Ce-TiO**_**2**_	**2.2%Ce-TiO**_**2**_
Crystal size (nm)	17.32	13.27	10.72	10.13
Crystal distortion	0.8217	0.9735	1.195	1.252

### SEM observations

**Figure 4:**
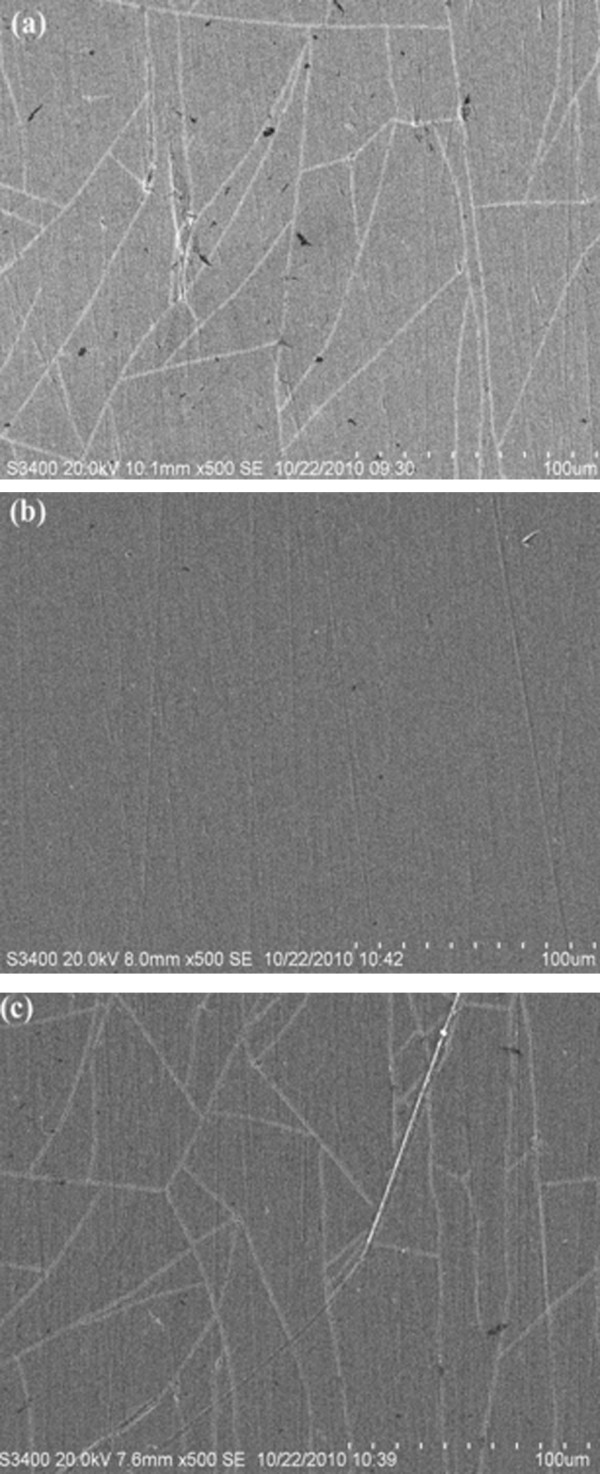
**SEM images of different Ce-TiO**_**2**_**coatings.** (**a**) pure TiO_2_, (**b**) 1.2%Ce-TiO_2_, and (**c**) 2.2%Ce-TiO_2_.

Figure 4 presents the SEM micrograph of the 316 L stainless steel coated with pure TiO_2_ film, 1.2% Ce-doped TiO_2_ film and 2.2% Ce-doped TiO_2_ film. The surface of the undoped coating contains some cracks and also a few pinhole-like defects because of the shrinkage occurring during the thermal treatment. The sample doped with 1.2% cerium ion content seems to be more uniform and crack-free. It is believed that after doping with cerium in the TiO_2_ coating, the growth of the crystal is restrained, and therefore, the size becomes much smaller and the distribution of the nanoparticles gets more even and compact. It can be inferred that the cerium ion-doped TiO_2_ film offers a more efficient protection for the metals as result of its smooth and uniform surface. The 2.2% Ce-doped coating has some cracks but fewer defects. It was attributed to the presence of too much cerium content in the coating.

### EIS analyses

**Figure 5:**
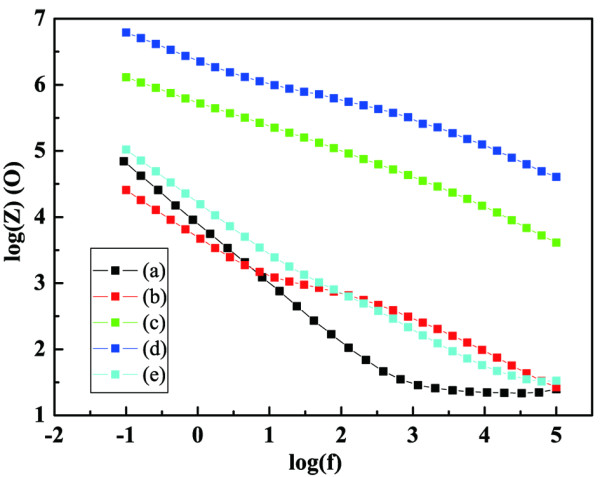
**EIS spectra for different Ce-TiO**_**2**_**coatings after 7 days of immersion.** (**a**) bare 316 L, (**b**) pure TiO_2_, (**c**) 0.2%Ce-TiO_2_, (**d**) 1.2%Ce-TiO_2_, and (**e**) 2.2%Ce-TiO_2_.

EIS was used to study the effect of cerium ion doping on electrochemical properties of the TiO_2_-coated 316 L in the absence of illumination. Figure 5 shows the impedance spectra of the bare 316 L and the coated 316 L samples after 7 days of immersion in the 3 wt% NaCl solution without photo effect.

It can be seen that 1.2% Ce-TiO_2_ coating exhibits the highest impedance values. By comparison, the impedance magnitude of 2.2% Ce-TiO_2_ coating decreases significantly after immersion, offering very little protection and behaving in a manner very similar to that of the uncoated sample.

**Figure 6:**
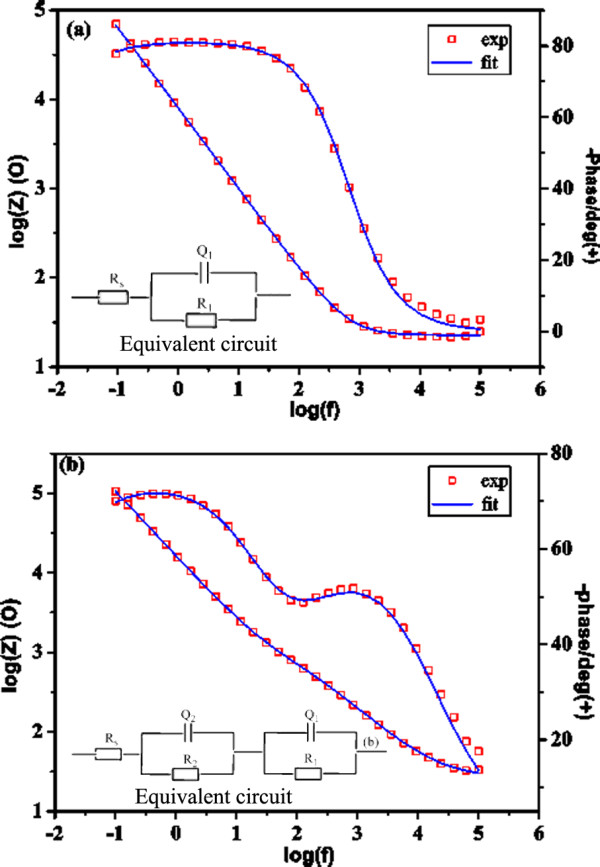
**Fitting curves for the impedance spectra.** (**a**) bare 316 L and (**b**) 2.2% Ce-TiO_2_ coating. Symbols are corresponding to the experimental data and *full lines* are for theoretical curves. Inset in panel (a) and (b) are the corresponding equivalent circuits used for numerical fitting of the EIS data

In order to get more detailed analyses of the corrosion protection properties attributed to different coatings, the impedance spectra were fitted using the appropriate equivalent circuit models, which were shown in the inset of Figure 6. The fitting curves for the impedance spectrum of bare 316 L and the coated 316 L were plotted as depicted in Figure 6, on which an excellent agreement between the experimental data and the theoretical curves can be obtained.

After 7 days of immersion, the impedance spectra show two typical time constants for the coated 316 L and only one time constant for the bare 316 L. Adopting the usual interpretation employed for coated metals, the time constant in the high frequency region is attributed to the sol–gel coating properties, and a pair of elements of *R*_2_ and *Q*_2_ in parallel represents the resistance and constant phase element (CPE). Here, CPE is commonly used to replace capacitance because it is hardly pure capacitance in the real electrochemical process and it is defined by admittance *Y* and power index number *n*, given by *Y* = *Y*_0_ (jω)^*n*^. This is a general dispersion formula, for *n* = 0 it stands resistance, while it is capacitance if *n* = 1. In all cases of the study, *n* is close to 1, representing a capacitive characteristic of the interfaces. The time constant at low frequency region is related to the properties of passive film present on the stainless steel surface, and a pair of elements of *R*_1_ and *Q*_1_ in parallel represents its resistance and CPE. This oxide film may be formed naturally and also results from the reaction with the solution, which penetrates the outer coating after long-time immersion. As for the bare stainless steel, the time constant can be attributed to the response of the passive film formed on the 316 L and *R*_s_ is the resistance of the solution.

Table 2 presents the corresponding electrochemical parameters obtained in the equivalent circuit. Usually, the uniform and thick coatings behave as an insulator with high resistances and low capacitances. It can be seen that the resistance in the high frequency region (*R*_2_) of 1.2% Ce-TiO_2_ coating is almost two orders of magnitude higher than that of undoped coating indicating a reduced accessibility of electrolyte due to its compact structure which is in agreement with the results from SEM observation. By comparison, 2.2% Ce-TiO_2_ gives lower coating resistance and higher capacitance. This means the corrosion protection property is not always proportional to the doping cerium content. It can be inferred that there is an optimum concentration for beneficial effects of cerium ion on corrosion resistance, while higher concentration of cerium ions in the sol–gel coating leads to the formation of a fragile film with poor barrier properties. Increase in the amount of cracks and defects in the sol–gel layer seem to be mainly responsible for this negative effect.

**Table 2:** EIS parameters in equivalent circuit for the bare and coated 316 L systems

**Samples**	***R***_**s**_**(Ω)**	***Q***_**2**_		***R***_**2**_**(Ω)**	***Q***_**1**_		***R***_**1**_**(Ω)**
		***Y***_**0**_	***n***		***Y***_**0**_	***n***	
Bare 316 L	31.22	―	―	―	9.984 × 10^−6^	0.8635	1.803 × 10^5^
TiO_2_	29.85	1.632 × 10^−5^	0.7882	8.797 × 10^2^	5.368 × 10^−5^	0.8053	3.177 × 10^5^
0.2%Ce-TiO_2_	30.63	1.738 × 10^−7^	0.7969	6.736 × 10^3^	7.309 × 10^−7^	0.8541	3.905 × 10^6^
1.2%Ce-TiO_2_	31.08	1.412 × 10^−8^	0.8376	4.853 × 10^4^	1.952 × 10^−7^	0.8835	4.581 × 10^7^
2.2%Ce-TiO_2_	29.92	1.979 × 10^−5^	0.7213	6.447 × 10^2^	1.355 × 10^−5^	0.8223	1.495 × 10^6^

Moreover, it is obvious that the resistance in the low frequency region (*R*_1_) of cerium ion-doped coatings is almost one or two orders of magnitude higher than that of the undoped coating, confirming higher corrosion protection for the substrate. The protective role of Ce ions is attributed to the precipitation of cerium hydroxides and oxides in the cathodic areas of the passive film. Such species can block the cathodic sites hindering the cathodic reaction and consequently, the corrosion rate.

### SEM-EDX analysis

**Figure 7:**
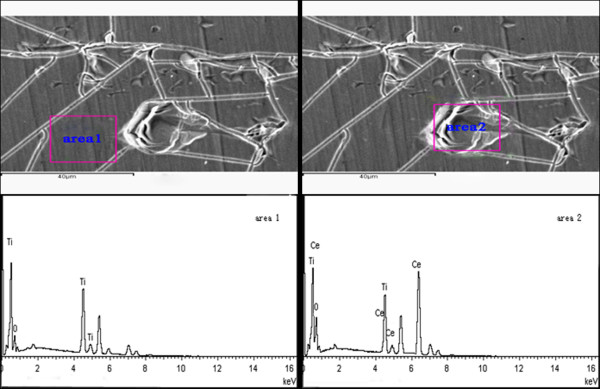
**EIS-EDX analysis of 1.2% Ce-TiO**_**2**_**coating after EIS test in 3 wt% NaCl solution.** Area 1 and area 2 on SEM micrographs mark the compact area and the defect of the coating respectively, where EDX tests were performed.

SEM-EDX is tested after EIS analysis in the 3 wt% NaCl solution. As can be seen from Figure 7, the cerium ions do not have a homogeneous distribution, and the Ce content is higher in the defect (marked with area 2) compared to that exists in the compact area (marked with area 1). The reason is the release of hydroxyl ions in the cathodic areas around the defect which leads to the precipitation of cerium hydroxides and oxides. The phenomenon further demonstrates some kind of self-healing effect provided by cerium ions in the specific areas.

## Conclusion

We have successfully prepared the smooth and compact cerium ion-doped nano-TiO_2_ coating on the 316 L stainless steel by the sol–gel and dip-coating techniques. The photogenerated cathodic protection property of the TiO_2_ coating is enhanced because of the higher separation efficiency of electron–hole pairs and higher photoelectric conversion efficiency obtained by doping with an appropriate amount of cerium ions in the coating. The 1.2% Ce-TiO_2_ coating with three layers exhibits the best photogenerated cathodic protection in the 3 wt% NaCl solution. Furthermore, the presence of cerium ions also improves the corrosion protection property of the nano-TiO_2_ coating without the photo effect attributed to the inhibiting and self-healing properties of cerium ions. The results reveal that there is an optimum concentration of cerium nitrate around an atomic ratio (Ce/Ti) of 1.2% for the coating with the best corrosion protection performance in the dark. The higher concentration could increase the amount of cracks and defects in the sol–gel layer and lead to a negative effect in the barrier properties of the coating. Consequently, the cerium ion-doped nano-TiO_2_ coating can not only provide a better photogenerated cathodic protection to a metal substrate in the presence of illumination but also act as a better anticorrosive barrier in the absence of illumination.

## Competing interest

The authors declare that they have no competing interests.

## Authors’ contributions

The work presented here was carried out in collaboration between all authors. SNL carried out the laboratory experiments and wrote the paper. QW and YW interpreted the results. TC analyzed the data and participated in the revision of the manuscript. ZHZ helped in drafting the manuscript. JJF designed methods and defined the research theme. All authors read and approved the final manuscript.
